# Reflections upon the Life of Medical Men

**Published:** 1882-05

**Authors:** 


### REFLECTIONS UPON THE LIFE OF MEDICAL MEN.

IT is painful to reflect that, after a long life devoted to a pursuit full of anxiety, responsibility and personal sacrifice, our career is arrested by disease or premature old age. It is a melancholy fact that very few in the profession of medicine reach that common allotment of "three score and ten." The reason is found in the painfully rigorous and exacting nature of the profession. Before the physician reaches his meridian, there can be seen the marks of corroding care deeply furrowed in his face, and evidences of premature descendence. He is made but too conscious of this in the absence of that vigor, elasticity, and physical energy evinced by others, no younger than himself, but in different positions. His long intercourse with affliction, and the painful contemplation of the sorrows of his fellow-men, tend to subdue the spirits and destroy their elasticity by the austere habits of his life. Thus physically and mentally he pays the penalty of his choice of a profession; honorable and exalted as it may be, when faithfully and honorably filled, it is nevertheless the most imperative and exacting of all others. He looks back upon his career, and in reflecting upon the scenes through which he has passed, the exposures he has suffered, the great injustice which too often he has been compelled to endure, and there is nothing but a deep sense of duty which he owed to benevolence and humanity which would induce him to retrace his history or undergo the untold sufferings, mental and physical, through which he has passed.

We have often looked into the deep fissures which care and anxiety had furrowed in the faces of those of our brethren who had hearts to feel, and whose long experience had put their feelings to the test, and wondered that even a smile could dispel the dark cloud which overcast their countenances. It did appear to me that I could see the working of a mind engaged in deep retrospection upon some scenes and incidents which memory, ever busy, was bringing into view. The thought of the dying struggles of a beautiful and innocent babe, an only child, the fondest object of a mother's love, comes athwart his memory, and a pang of mental suffering contracts his countenance, and an irrepressible sigh is involuntarily drawn. The recollection of the last moments of a young and an affectionate wife, over whose couch he had hung for days and nights together, tortured by an intensity of anxiety which no language can describe, and under a responsibility which was most crushing to his moral powers. But death had triumphed over the resources of art and science. These, with numberless other trials, are ours to encounter, and the consequence is, that he soon wears away, and finally sinks under the accumulated weight of care and labor which, anticipating the ordinary and natural law of decline and loss, grind him down enervates his physical man, and soon he is driven to his repose in the cold and silent grave. We have been led to these reflections upon the perusal of a letter from a distinguished medical friend, and one for whom we entertain the most profound esteem, respect and affection. Like ourselves, he has passed over a rugged pathway in his professional career, and like ourselves too, he can look back in the past upon a picture whose back ground is cast in melancholy shades, with here and there a brighter object in relief. We will give his own thoughts in the following beautiful and touching paragraph.

"Ill health and premature decrepitude have necessarily drawn me into retirement, from the field of general practice. Although I may be said to be in the prime of life, yet such is the prostrated condition of my physical powers that I am compelled to relinquish a profession which I could not, under other and more favorable circumstances, leave sooner than a fond mother could forsake her sucking child. I have thought some of visiting your city to avail myself of such advice and counsel as the Faculty could give, in order to improve my health, if thought by yourself and colleagues possible. But for my part I am quite discouraged and depressed, and I fear there is not sufficient recuperative energy left me to rally under the most skillful treatment, having tried faithfully every remedy offering the least hope of success.

"I have now nearly crossed the 'stormy sea' of professional life, and may be said to be standing upon the other shore, awaiting the call of the 'Great Physician' to depart hence, and thereby escape the afflictions of the mortal body for a life everlasting, free from sorrow, pain and suffering. In reviewing my past life as a member of an honorable profession, I am consoled in the belief that in my limited and humble sphere I never knowingly did anything calculated to impair the credit, or debase the dignity of the profession; and although I am in a great measure retired and buried from the medical world, I shall always look after the honor and purity of the faculty, as I would watch after and protect the honor and welfare of the wife of my youth."

Such sentiments are worthy of the noble, generous, and lofty mind that conceived them, and should be adopted by every member of the profession. For our part, we could not subdue a rising emotion which swelled our bosom and moistened the eye with a sympathising tear, when reading the letter containing the above extract, and of offering up a silent prayer that he might be relieved of his sufferings and speedily return to the enjoyment of health, and be allowed many years to live. We cannot well spare such men from among us, even if they live in retiracy, for their moral influence must be felt, not only in the community, but particularly in the professional ranks.—*Iowa Med. Jour.*

